# Rare Association of Lingual Hemiatrophy With Meningiomatosis: A Case Report

**DOI:** 10.7759/cureus.95334

**Published:** 2025-10-24

**Authors:** Fatima Zeineddine, Abbas Rachid, Mohammad Rida Noureddine, Assaad Mohanna, Oussama Rihan

**Affiliations:** 1 Radiology, American University of Beirut Medical Center, Beirut, LBN; 2 Internal Medicine, Lebanese University, Beirut, LBN; 3 Otolaryngology, Bahman University Hospital, Beirut, LBN; 4 Radiology, Bahman University Hospital, Beirut, LBN

**Keywords:** hypoglossal canal, hypoglossal nerve, meningioma, meningiomatosis, schwannoma

## Abstract

Hypoglossal nerve (cranial nerve XII) palsy is a rare condition, especially when isolated, and may arise from various etiologies, including tumors along its course. Among the rarest causes are meningiomas involving the hypoglossal canal. Meningiomatosis, the presence of multiple meningiomas throughout the central nervous system, is an uncommon entity that further complicates diagnosis. We report a case of a 64-year-old woman presenting with progressive right-sided tongue atrophy and deviation, without associated dysphonia or dysphagia. CT and MRI revealed fatty infiltration of the right hemitongue and demonstrated more than 10 intracranial meningiomas, including one involving the right hypoglossal canal, associated with bone remodeling. Imaging features, including the dural tail sign, homogenous enhancement, and adjacent bony changes, favored a meningioma over other differential diagnoses, such as schwannoma. The co-occurrence of meningiomatosis and isolated hypoglossal nerve palsy in a patient without neurofibromatosis type 2 is exceedingly rare. This case highlights the critical role of advanced imaging in localizing cranial neuropathies and underscores the need to consider meningiomatosis in the differential diagnosis of isolated hypoglossal nerve palsy.

## Introduction

Unilateral tongue hemiatrophy, often accompanied by fatty replacement, is a hallmark of hypoglossal nerve (cranial nerve XII) palsy, a purely motor disorder of the cranial nerve. Isolated hypoglossal paralysis is rare and may result from a wide range of etiologies, including vascular, inflammatory, traumatic, or space-occupying lesions along the nerve’s complex anatomical course. Thorough evaluation is therefore required from its origin in the medulla, through the ventral brainstem, the hypoglossal canal where it exits the skull, and its extracranial segments within the carotid space and sublingual region [1]. Anatomically, the hypoglossal nerve is divided into intracranial segments (nuclear, cisternal, and skull base) and extracranial segments (carotid and sublingual) [2]. Tumors are the most common cause of unilateral hypoglossal nerve palsy, accounting for nearly 50% of cases, with the cisternal and skull base regions most frequently involved. The differential diagnosis in these regions includes meningiomas, schwannomas, paragangliomas, chordomas, metastases, leptomeningeal disease, and nasopharyngeal carcinoma [3,4]. Although meningiomas are common intracranial tumors, those originating within the hypoglossal canal are extremely rare, with only a few cases reported in the literature [5]. Even more uncommon is meningiomatosis, a condition characterized by the presence of multiple meningiomas throughout the central nervous system, accounting for approximately 2% of meningioma cases. Its etiology remains unclear in many cases but may be sporadic, radiation-induced, or associated with genetic conditions such as neurofibromatosis type 2 (NF2) [6,7].

Here, we present an exceedingly rare case of meningiomatosis involving the hypoglossal canal, manifesting as symptomatic unilateral tongue atrophy. This case highlights the diagnostic utility of CT and MRI in evaluating hypoglossal nerve lesions and underscores the importance of considering rare tumor variants in the differential diagnosis.

## Case presentation

**Figure 1:**
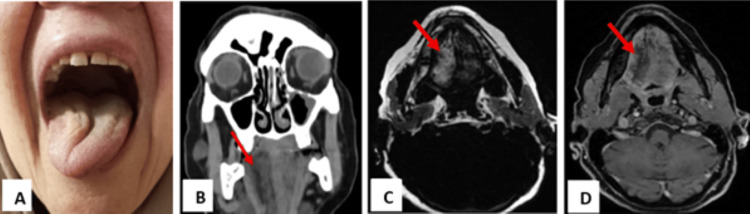
(A) Physical examination showing hemiatrophy of the tongue on the right side, and the inability to completely deviate the tongue toward the left side on protrusion. (B) Coronal reconstruction CT scan. (C) Axial T1 Dixon fat-only sequence in MRI. (D) Axial T1 fat-saturated imaging with contrast administration, showing fatty denervation of the right hemitongue (red arrow). CT scan: computed tomography scan; MRI: magnetic resonance imaging

A 64-year-old female presented with several months of difficulty moving her tongue. She had no prior history of malignancy, head or neck surgery, or radiation therapy. On physical examination, there was visible hemiatrophy of the right side of the tongue, and deviation was noted toward the right upon protrusion (Figure 1A). The remainder of the neurological examination was unremarkable.

**Figure 2:**
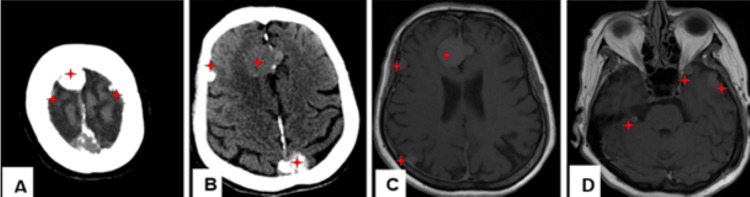
(A) and (B) Axial CT scan, (C) and (D) MRI axial T1W after IV gadolinium contrast administration, showing multiple extra-axial supra- and infratentorial lesions throughout the brain, the majority being calcified, and enhancing after IV gadolinium administration, compatible with mengingiomatosis (red asterisk). CT: computed tomography; MRI: magnetic resonance imaging; T1W: T1-weighted; IV: intravenous

Computed tomography (CT) of the brain and neck was performed, showing hemiatrophy of the right half of the tongue with fatty infiltration, confirming right hypoglossal nerve palsy (Figure 1B). In addition, multiple (more than 10) extra-axial supra- and infratentorial lesions throughout the brain, with the majority being calcified, are compatible with meningioma (Figures 2A-2B).

**Figure 3:**
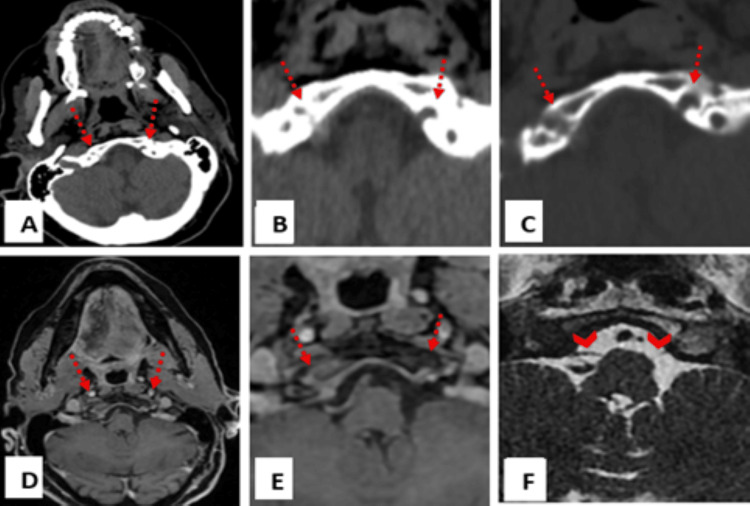
(A) Axial CT scan, (B) with magnified view: an intracranial extra-axial lesion at the level of the cisternal segment of the hypoglossal nerve with slight extension into the right hypoglossal canal. (C) Magnified view, axial CT scan bone window: cortical bone erosion of the right hypoglossal canal (right dashed arrow) compared to the intact canal on the left side (left dashed arrow). (D) T1 fat-saturated MR imaging with contrast administration, (E) with magnified view: 6 x 13 mm extra-axial enhancing lesion with a “dural tail” sign at the level of the cisternal segment of the right hypoglossal nerve extending to the right hypoglossal canal, with overlying bone edema and enhancement. (F) Magnified view, axial FIESTA MR imaging: thickening of the cisternal segment of the right hypoglossal nerve (right arrow head) compared to the intact and thin nerve on the left side (left arrow head). FIESTA MR: fast imaging employing steady-state acquisition magnetic resonance

Detailed examination of the right hypoglossal nerve revealed an extra-axial lesion within its cisternal segment, with extension into the right hypoglossal canal (Figures 3A-3B). Associated bone changes were observed at the level of the hypoglossal canal (Figure 3C).

Magnetic resonance imaging (MRI) with intravenous gadolinium was subsequently performed for further evaluation. Axial T1-weighted Dixon fat-only and contrast-enhanced fat-saturated sequences confirmed fatty denervation of the right hemitongue (Figures 1C-1D). Multiple enhancing extra-axial lesions were again noted throughout the brain, compatible with meningiomas, demonstrating homogeneous post-contrast enhancement (Figures 2C-2D). Notably, a 6 × 13 mm enhancing lesion with a characteristic "dural tail" was seen at the cisternal segment of the right hypoglossal nerve, extending into the hypoglossal canal and associated with adjacent bone edema and enhancement (Figures 3D-3E). An axial FIESTA sequence demonstrated thickening of the right hypoglossal nerve in its cisternal segment (Figure 3F).

Given the lesion’s deep location and the presence of multiple meningiomas, a conservative management approach was selected following discussion with the patient. She was advised to undergo close clinical and radiological follow-up.

## Discussion

The hypoglossal nerve (CN XII), a purely motor cranial nerve supplying the tongue, originates in the medulla and courses through the hypoglossal canal to the sublingual region, defining intracranial (nuclear, cisternal, skull base) and extracranial (carotid, sublingual) segments. Rare palsies present variably: supranuclear lesions cause contralateral tongue deviation without atrophy, while lower motor neuron lesions in segments such as the cisternal, carotid, or sublingual lead to ipsilateral deviation with atrophy and fasciculations. Involvement of infrahyoid strap muscles in carotid and sublingual palsies may also manifest as a hoarse voice and impaired swallowing, providing further localizing clues [2]. In our case, right tongue atrophy with ipsilateral deviation, absent voice or swallowing problems, points to a pathology specifically in the right hypoglossal nerve's cisternal or skull base segment.

When radiologists observe tongue ptosis and atrophy on CT or MRI, it strongly suggests potential hypoglossal nerve pathology. Comprehensive imaging, from the medulla to the sublingual space, is then essential to pinpoint the cause. Knowledge of common pathologies affecting each nerve segment aids in differential diagnosis. Both MRI and CT play vital and complementary roles. In acute stages, denervation edema appears as increased T2 signal and gadolinium enhancement on MRI. Chronic denervation is characterized by reduced muscle bulk, fatty infiltration (as indicated by hypoattenuation on CT and T1 shortening on MRI), and ipsilateral volume loss. MRI is preferred for soft tissue contrast and direct nerve visualization, especially for cisternal and skull base segments, as in our case, gadolinium-enhanced T1 gradient echo sequences and heavily T2-weighted SSFP sequences (FIESTA) are particularly useful for identifying the intracanalicular nerve and for detecting small tumors. However, CT is the best modality for examining the bony hypoglossal canal for fractures, cortical destruction, or remodeling [8].

Isolated hypoglossal neuropathy is rare, and when primary malignancy is excluded, meningiomas and schwannomas are among the primary differential diagnoses for cisternal or intracanalicular lesions [9-12]. Although both tumors are uncommon at this site, meningiomas arising within the hypoglossal canal are exceedingly rare. Differentiating these lesions radiologically can be challenging, especially due to the lack of specific studies focused on this region. However, features extrapolated from more common locations, such as the cerebellopontine angle (CPA) and internal auditory canal (IAC), can aid differentiation.

Meningiomas are often characterized by a broad dural base, the presence of a “dural tail” (a tapering, enhancing edge of dura seen in over 50% of cases), and associated bone changes such as hyperostosis or erosion. Intratumoral calcification is also more common in meningiomas. These features are well-depicted on CT, though they may also be appreciated on MRI. In contrast, schwannomas are typically well-circumscribed and globular and may exhibit cystic degeneration or necrosis. They rarely demonstrate a dural tail or bony hyperostosis [13-15]. In our case, the lesion demonstrated a dural tail, enhancement, and associated bone remodeling of the hypoglossal canal, features more characteristic of a meningioma. However, histopathological confirmation remains the gold standard.

What distinguishes our case is the co-occurrence of meningiomatosis and hypoglossal nerve palsy, an exceptionally rare presentation. The patient exhibited more than 10 intracranial meningiomas in both supra- and infratentorial compartments, one of which was localized to the right hypoglossal canal and responsible for the clinical symptoms. While meningiomas and schwannomas causing isolated hypoglossal nerve palsy are reported in the literature, involvement of the hypoglossal nerve in the context of meningiomatosis is extraordinarily rare. Previous reports of cranial nerve involvement in meningiomatosis have typically included the oculomotor or optic nerves, often through mass effect [7,16,17].

Importantly, our patient did not meet clinical or radiological criteria for neurofibromatosis type 2 (NF2), a known cause of multiple meningiomas. The occurrence of such widespread lesions in the absence of NF2 further emphasizes the unique nature of this case. It highlights the importance of detailed imaging assessment of the entire hypoglossal nerve course in patients with tongue hemiatrophy, especially when uncommon tumor variants such as meningiomatosis are involved.

## Conclusions

This case report documents an unusual presentation of hypoglossal nerve palsy caused by a cisternal meningioma extending into the hypoglossal canal in a patient with meningiomatosis. The clinical and imaging features enabled accurate localization and diagnosis without the need for biopsy. The absence of neurofibromatosis or prior radiation therapy adds to the rarity of this case. A comprehensive MRI and CT evaluation of the entire hypoglossal nerve pathway is essential when patients present with isolated tongue atrophy. Furthermore, this case reinforces the importance of including meningiomatosis in the differential diagnosis for isolated cranial nerve palsies, even in sporadic settings.

## References

[1] Mahadevappa K, Chacko T, Nair AK (2012). Isolated unilateral hypoglossal nerve palsy due to vertebral artery dissection. Clin Med Res.

[2] Ragittaran J, Kamalasanan A, White RD, Sudarshan T (2025). Imaging of hypoglossal palsy: a pictorial synopsis. Clin Radiol.

[3] Weindling SM, Goff RD, Wood CP, DeLone DR, Hoxworth JM (2016). Is hypoglossal nerve palsy caused by craniocervical junction degenerative disease an underrecognized entity?. AJNR Am J Neuroradiol.

[4] Calzada G, Isaacson B, Yoshor D, Oghalai JS (2007). Surgical approaches to the hypoglossal canal. Skull Base.

[5] Shibao S, Yoshida K, Sogano J, Mizutani K, Tomita H (2022). Meningiomas involving the hypoglossal canal: a case report and literature review. NMC Case Rep J.

[6] Mannatrizio D, Testini V, Fascia G, Guerra FS, Masino F, Bellitti R, Guglielmi G (2025). Multifocal meningiomatosis: a case study highlighting diagnostic and monitoring challenges. Acta Biomed.

[7] Fahmi M, Widhianingsih NL (2024). Acquired ptosis in patient with suspect meningiomatosis. Magna Neurologica.

[8] Gorolay VV, Tran NA, Tade R, Baugnon K, Aiken A, Wu X (2023). The ptotic tongue-imaging appearance and pathology localization along the course of the hypoglossal nerve. Neuroradiology.

[9] Skandhan AK (2018). Tongue hemi atrophy-hypoglossal neurolemmoma. Int J Radiol Radiat Ther.

[10] Zurale MM, Patil A (2023). A rare case of hypoglossal nerve schwannoma presenting as hemiatrophy of the tongue: a case report. Ann Indian Acad Neurol.

[11] Heda S, Karthik DK, Rao ES, Deshpande A (2018). Hypoglossal canal schwannoma causing isolated left 12th cranial nerve palsy. BMJ Case Rep.

[12] O'Gorman C, Willis A (2019). An uncommon cause of isolated hypoglossal nerve palsy: a case report. J Ir Dent Assoc.

[13] Lalwani AK, Jackler RK (1993). Preoperative differentiation between meningioma of the cerebellopontine angle and acoustic neuroma using MRI. Otolaryngol Head Neck Surg.

[14] Molga-Magusiak M, Laskus A, Karchier E (2017). Meningoma located primarily within the internal auditory canal - a case report. Pol Otorhino Rev.

[15] Watts J, Box G, Galvin A, Brotchie P, Trost N, Sutherland T (2014). Magnetic resonance imaging of meningiomas: a pictorial review. Insights Imaging.

[16] Navarro-Olvera JL, Parra-Romero G, Carrillo-Ruiz JD, Aguado-Carrillo G, Hernández-Valencia AF (2021). Resection of meningiomas in a different location (sphenoid wing and tuberculum sellae) through a single craniotomy report of a case and review of the literature. Cir Cir.

[17] Sharma S, Sharma P, Kumar A (2021). Diffuse meningiomatosis without neurofibromatosis: a rare diagnosis with atypical presentation. Indian J Radiol Imaging.

